# In vitro prospective healthy and nutritional benefits of different *Citrus* monofloral honeys

**DOI:** 10.1038/s41598-023-27802-1

**Published:** 2023-01-19

**Authors:** Florinda Fratianni, Giuseppe Amato, Antonio d’Acierno, Maria Neve Ombra, Vincenzo De Feo, Raffaele Coppola, Filomena Nazzaro

**Affiliations:** 1 Institute of Food Sciences, CNR-ISA, Avellino, Italy; 2 Department of Pharmacy, University of Salerno, 84084 Fisciano (Salerno), Italy; 3 Department of Agriculture, Environmental and Food Sciences, University of Molise, 86100 Campobasso, Italy

**Keywords:** Biochemistry, Chemical biology, Microbiology, Health care

## Abstract

We studied the total polyphenols, flavonoids, vitamin C, the antioxidant and anti-inflammatory activity of six *Citrus* monofloral honey, and the in vitro inhibitory effect against cholinesterases and tyrosinase. Finally, we assessed their effect against the biofilm of some pathogenic bacteria. Lime honey showed the best antioxidant activity and the highest content of polyphenols and vitamin C. Lemon and tangerine honey contained almost exclusively flavonoids. Lemon honey better preserved the bovine serum albumin against denaturation (IC_50_ = 48.47 mg). Honeys inhibited acetylcholinesterase, butyrylcholinesterase, and tyrosinase up to 12.04% (tangerine), 19.11% (bergamot), and 94.1% (lemon), respectively. Lime and clementine honey better inhibited the *Listeria monocytogenes* biofilm. Bergamot honey acted mainly against the *Staphylococcus aureus* and *Acinetobacter baumannii* biofilm; bergamot and tangerine honey inhibited the *Pseudomonas aeruginosa* biofilm particularly. Bergamot, clementine, and tangerine honey acted against *Escherichia coli* sessile cell metabolism. This *Citrus* honey exhibited in vitro prospective health benefits and is applicable for future in vivo studies.

## Introduction

Honey represents the main product of the apiculture, which history coincides with that of the mankind. Its benefits were indicated in the medicine of the Mediterranean area, and described from the Egyptian medicine, more than 5,000 years ago, for a broad variety of therapeutic application^1^. The benefits of honey can be attributed to its different bioactive molecules, which percentage varies among different kinds of honey. Polyphenols, flavonoids and vitamins represent some of the most important bio-components present in the composition of the honey^2^. Polyphenols, one of the major class of naturally occurring organic compounds defined by multiples of phenol units, consist of flavonoids and non-flavonoids. Honey contains a wide range of both flavonoids and non-flavonoids. Vitamin C, with group B-vitamins is one of the major vitamins found in honey. Honey exhibits significant antimicrobial^3,4^, and antiviral^5^ property. Furthermore, it can act as an antioxidant^6^, anti-inflammatory^7^, anticarcinogen^8^, antimutagenic^9^, and immune-boosting^10^ agent. Its cardio protective^11^, hypocholesterolemic^12^, and hypoglycemic^13^ effects are recognized too. In last years, the in vitro and in vivo studies, corroborated the protective effect of the honey in the neurological disorders, which represent one of the major health issues mainly in the western countries^14^. Among neurological disorders, neurodegenerative diseases (NDDs) such as Alzheimer’s disease (AD), and Parkinson’s disease (PD), are the most commonly prevalent, and represent a consequence of neuronal death occurring in different parts of the brain and the Central Nervous System^15^, mainly manifesting in elderly people^16^. Neurodegenerative diseases can see the involvement of some enzymes, such as acetylcholinesterase (AChE), butyrylcholinesterase (BChE), and tyrosinase. AChE and BChE are the enzymes responsible for neurotransmitter degradation^17^. Their inhibition can be considered possible prevention and treatment of the Alzheimer’s disease^18^. The excessive presence of tyrosinase seems particularly linked to Parkinson's disease, as it increases intracellular dopamine, produces a large amount of melanin, and consequent cell death^19^. Several NDDs can be related also to the involvement of neuro-inflammation, which determine neurodegeneration^20^. In addition, oxidative stress, caused by an amassing of free radicals and lessening of antioxidants can determine the nerve death^21^.

In recent years, a certain number of bacterial infections was connected to an increased risk of neurodegeneration^22^; the formation of bacterial biofilms also determines the bacterial production of amyloid structures, used to fortify the biofilm matrix, that, in clinical, are associated with protein misfolding and neurodegenerative diseases^23^. Increasing the levels of antioxidants can be beneficial against these neurodegenerative diseases. Polyphenols, flavonoids and vitamins, such as vitamin C, present also in the honey, act as antioxidants and anti-inflammatory agents^24,25^. These molecules can protect neurons against damage, enhance neuronal function and augment regeneration, with the consequence to avoid or limit neurotoxicity, and better regulate neuronal signaling pathways^26^. Starting from all such considerations, this research evaluated the content of total polyphenols, flavonoids and vitamin C of the honey of six species of *Citrus* (tangerine, lime, lemon, bergamot, orange, and clementine). Furthermore, some biological properties, such as the inhibitory potential against cholinesterases and tyrosinase, the antioxidant and the anti-inflammatory activity exhibited by these types of honey were studied. Finally, we evaluated the ability of the *Citrus* monofloral honeys to act on the biofilm formation capacity exhibited in vitro by *Acinetobacter baumannii*, *Escherichia coli*, *Listeria monocytogenes*, *Pseudomonas aeruginosa* and *Staphylococcus aureus*, which are some of the bacteria whose presence or over-presence can contribute to the triggering of NDDs.

## Materials and methods

Different *Citrus* types of organic monofloral honey: bergamot (*Citrus x bergamia* Risso & Poit., company Blanco 1837, “Agrisicilia”, region of provenience: Sicily, Southern Italy), lemon [*Citrus limon* (L.) Burm. f., company “l’Ape & l’Arnia, Azienda Iacovelli”, region of provenience: Marche, Central Italy], lime [*Citrus aurantiifolia* (Christm.) Swingle, company “Bendis honey” country of provenience: Romania], orange [*Citrus sinensis* (L.) Osbeck], company “Thun”, region of provenience: Sicily, Southern Italy), tangerine (*Citrus reticulata* Blanco, company Blanco 1837, “Agrisicilia”, region of provenience: Sicily, Southern Italy), were used for our experiments. Three packages of honey were used, making sure they were from the same year of production. The companies provided the requested analyses before placing them on the market. No sample showed crystallization. Honey was stored in the dark until the analyses; then, samples were suspended by a mixer in deionized water and phosphate buffer solution (1 g of honey dissolved in 4 ml of solution). Mixture was filtered (0.45 µm, Millipore, Milano, Italy) and subjected to the biochemical analyses and the microbial tests.

### Total polyphenol content

Total polyphenols content (TPC) was assessed at room temperature with the Folin–Ciocalteu phenol reagent^27^. The absorbance was measured by a UV/Vis spectrophotometer at 760 nm (Cary Varian, Palo Alto, CA, USA). Results were expressed in terms of µg of gallic acid (used as standard) equivalents (GAE)/g of honey ± standard deviation (SD). The concentration range for the standard curve was produced between 34.02 and 340.2 µg.

#### Total flavonoids content

The total flavonoid content was determined following the method described by Ombra and coworkers^28^, adapted as follows: each sample of honey solution (50 µl) was mixed with 215 µL of 5% NaNO_2_ and incubated for 5 min. Then, added 15 µl of 10% anhydrous AlCl_3_ were added in each sample, which was incubated for 1 min. Lastly, 220 µl of NaOH 1 M were added, and the volume was taken up 1 ml with deionized water. One hundred and fifty µl of this reaction were read at 510 nm with a microplate reader. All samples were analyzed in triplicate, and results were expressed as µg rutin equivalent (RE) g^−1^ of honey ± SD.

#### Ascorbic acid content

The ascorbic acid was determined following the method of the reduction of the dye 2,6-dichlorophenolindophenol (DCPIP)^29^, with some modifications. A solution of 0.3 mg ml^−1^ DCPIP was incorporated in each sample containing ascorbic acid in 2.5% metaphosphoric acid and citrate-acetate buffer. The absorbance was measured after 45 s at 520 nm. Results were expressed as µg of ascorbic acid/100 g of the sample ± SD.

#### Antioxidant activity

The antioxidant activity was calculated by the azino-bis (3-ethylbenzothiazoline-6-sulfonic acid (ABTS) test^30^. The ABTS diammonium salt, potassium persulfate (dipotassium peroxidisulfate), 6-hydroxy 2,5,7,8-tetramethylchroman-2-carboxylic acid (Trolox), and HPLC grade methanol were obtained from Sigma-Aldrich Milano, Italy. Trolox, 2.5 mM in methanol prepared day-to-day, was used as antioxidant stock standard. The ABTS and potassium persulfate were dissolved in distilled water to a final concentration of 7 and 2.45 mM, respectively, and mixed. The mixture was kept in the dark at room temperature for 16 h before its use to produce the ABTS radical (ABTS^+^). The ABTS radical solution was diluted using distilled water to have an absorbance of 1.00 at 734 nm. Samples (final concentrations 0.0001–0.0100 mg/ml) or Trolox standard (final concentration 0–20 mM) were added to the diluted ABTS^+^ solution; the absorbance was read 6 min after mixing by a UV/Vis spectrophotometer (Cary Varian, Palo Alto, CA, USA). Determinations were performed in triplicate and results expressed as the samples μmol Trolox equivalent antioxidant capacity (TEAC) ± SD.

#### Inhibitory effect of the honey against the bovine serum albumin degradation

The assay of the inhibition of serum bovine albumin denaturation was used to evaluate the in vitro anti-inflammatory activity following the method of Fratianni et al.^31^. Briefly, a stock solution of 0.5% (w/v) bovine serum albumin (BSA, 96% purity, Sigma, Milano, Italy) in 0.05 M tris–phosphate buffer saline solution was prepared and the pH was adjusted to 6.5 using glacial acetic acid. The reaction mixture (5 ml) comprised 0.2 ml of BSA, 2.8 ml of phosphate tris–phosphate buffer saline solution, and 2 ml of varying amounts (5, 10, 20, 30 μg) of honey. One ml of BSA containing methanol was used as a control. After 5 min of heating at 72 °C, samples were cooled. The absorbance was calculated at 660 nm by a UV/Vis spectrophotometer (Cary Varian, Palo Alto, CA, USA). The extract concentration for the 50% inhibition (IC_50_) of the BSA denaturation was determined compared with the control and using diclofenac sodium (1 mg/ml) as the positive control.

#### Cholinesterase inhibitory activities

The inhibitory activity on acetylcholinesterase (AChE) and butyrylcholinesterase (BChE) was determined by the spectrophotometric method of Ellman et al.^32^. Acetylcholine (ACh) was the substrate to assay the inhibition of AChE. The reaction mixture contained 550 μl of sodium phosphate buffer 0.1 M (pH 8.0); 50 μl of the honey sample, and 5 to 20 ng of AChE (from *Electrophorus electricus* 1,000 units/mg), which were mixed and incubated for 15 min at room temperature. All samples and the positive control (galantamine, Sigma Aldrich, Milano, Italy) were dissolved in 10% dimethyl sulfoxide (DMSO). The hydrolysis of ACh was monitored following the formation of the yellow 5-thio-2-nitrobenzoate anion at 412 nm for 10 min, which resulted from the reaction of 5,5-dithio-bis-(2-nitrobenzoic acid (DTNB, 10 µl) with acetylcholine (10 μl), released by the enzymatic hydrolysis of ACh. The inhibitory activities on BChE was measured using butyrylthiocholine (BCh) as substrate. The reaction mixture contained: 550 μl K-phosphate buffer 0.1 M (pH 7.0), 50 μl of the sample, and an amount of equine serum BChE (≥ 10 units/mg protein) ranging from 10 to 50 ng. All ingredients were mixed and incubated for 15 min at room temperature. Then, the substrate BCh and the DTNB were added. The hydrolysis of BCh was monitored at 412 nm, following the formation of the yellow 5-thio-2-nitrobenzoate anion for 10 min, resulting from the reaction of DTNB (10 μl) with butyrylcholine (10 μl), released by the enzymatic hydrolysis of BCh. All reactions were performed in triplicate in 96-well microtiter plates. Percent inhibition was calculated using the equation:

$$\% \;{\text{inhibition}} = 1 - ({\text{B}} - {\text{b}}) \times 100/({\text{A}} - {\text{a}})$$

where B is an initial enzyme reaction with sample, and b is an initial reaction with sample but without enzyme A is an initial reaction with enzyme, a is an initial reaction without enzyme. Determinations were performed in triplicate and results expressed as the mean ± SD. The AChE and BChE inhibitory activities were also expressed in terms of the IC_50_ value (mg required to inhibit at 50% the hydrolysis of the substrate).

#### Tyrosinase inhibition assay

The tyrosinase inhibition test was performed as described by Khatib et al.^33^, with minor modifications. Honey samples were diluted (1:1) in dimethyl sulfoxide (DMSO). The reaction mixture was created by loading the phosphate buffer (70 μl, pH 6.8), (10 units/ml), and the sample directly on the 96 microtiter plate; after a five min-incubation at 37 °C, L-tyrosine (or 0.5 mM l- dioxyphenylalanine, (l-DOPA) was added, and the plate was immediately read at 492 nm. After incubation at 37 °C for 10 min, the optical density of samples was measured at 475 nm. Kojic acid was the positive control; phosphate buffer was the blank. The percentage of inhibition was calculated following the formula:

$$\% \;{\text{I}} = [({\text{Ab}} - {\text{As)}}/{\text{Ab}} \times 100]$$

where Ab: absorbance of the blank sample at T10; As: sample absorption at T10—sample absorbance at T0 (T0: beginning; T10: 10 min). Determinations were performed in triplicate and results expressed as the mean ± SD. The inhibitory activity on tyrosinase was also evaluated in terms of IC_50_, that is the concentration of samples giving a 50% inhibition of the tyrosinase activity. Such evaluation was performed by interpolation of the concentration–response curves. All tests were performed in triplicate for each sample and for the control.

### Antibacterial activity

#### Microorganisms and culture conditions

*Acinetobacter baumannii* (ATCC 19,606), *Escherichia coli* (DSM 8579), *Pseudomonas aeruginosa* (DSM 50,071), *Listeria monocytogenes* (ATCC 7644), and *Staphylococcus aureus* subsp. *aureus* Rosebach (ATCC 25,923) were the test bacterial strains used in our experiments. Before the antimicrobial assays, they were cultured in Luria Broth for 18 h at 37 °C (*A. baumannii* was grown at 35 °C) and 80 rpm (Corning LSE, Pisa, Italy).

### Minimal inhibitory concentration (MIC)

The resazurin microtiter-plate assay evaluated the MIC^34^. The tests were performed in flat-bottomed 96-well microtiter plates incubated at 37 °C for 24 h, except for *A. baumannii*, grown at 35 °C under the same conditions. The MIC value was revealed by the color change from dark purple to colorless. Determinations were performed in triplicate and results expressed as the arithmetic mean ± standard deviation.

### Inhibition of biofilm formation

The capacity of the honey to influence the bacterial biofilm formation was evaluated in flat-bottomed 96-well microtiter plates (Falcon, VWR International, Milano, Italy)^27^. Before the test, the overnight bacterial cultures were adjusted to 0.5 McFarland with fresh culture broth. Then, in each well 10 µl of the bacterial cultures, 10 µg/ml and 20 µg/ml of each honey- and Luria–Bertani broth (LB, Sigma Aldrich Italia, Milano, Italy) were brought to have a final volume of 250 µl. Microtiter plates were covered with parafilm tape to preclude the evaporation of material included in the wells and incubated for 48 h at 37 °C (plates containing *A. baumannii* were incubated at 35 °C). After removing the planktonic cells, the attached cells were lightly washed twice with sterile phosphate buffered saline (PBS), which was discarded, leaving the plates kept for 10 min under the flow laminar hood. Two hundred µl of methanol were included in each well and retained for 15 min for the fixation of the sessile cells. Methanol was discarded, and each plate was left to let the dryness, then we added 200 µl of 2% w/v crystal violet solution to each well. After 20 min, the staining solution was discarded, and the plates were lightly washed with sterile PBS and left to dry. The bound dye was released by adding 200 µL of glacial acetic acid 20% w/v. The absorbance was measured at 540 nm. The percent value of adhesion was calculated with respect to the control (formed by the cells grown without the presence of the samples, which inhibition rate was assumed as 0%). Triplicate tests were done, taking the average results for reproducibility, and results were expressed as the mean ± SD.

### Metabolic activity of biofilms

The effect on the metabolic activity of the biofilm was evaluated through the 3-(4,5-dimethylthiazol-2-yl)-2,5-diphenyltetrazolium bromide (MTT) colorimetric method^27^ using 96-well microtiter plates. The overnight bacterial cultures were adjusted to 0.5 McFarland, and the plates, with 10 µg/ml or 20 μg/mL of honey and Luria broth up to 250 μl, were prepared as previously described. After 48 h total of incubation, bacterial suspension, representing the planktonic cells, was removed, and 150 µl of PBS and 30 µl of 0.3% of MTT (Sigma, Milano, Italy) were added, keeping microplates at 37 °C (except than *A. baumannii*, incubated at 35 °C). The MTT solution was removed after two h, and the plates were washed twice with 200 µl sterile physiological solution. Next, 200 µl of DMSO were added, leading to the formazan crystals' dissolution, measured at 570 nm after two h. Triplicate tests were done, taking the average results for reproducibility, and results were expressed as the mean ± SD.

### Statistical analysis

Data were expressed as the mean ± SD of three experiments and statistically analyzed using a two-way ANOVA followed by Dunnett’s multiple comparison test, at the significance level of *p* < 0.05, using GraphPad Prism 6.0 (GraphPad Software, Inc., San Diego, California).

## Results and discussion

Since ancient times, honey has traditionally been a supporting factor in medical treatments. In recent years, some papers reported the protective effect of honey against some neurodegenerative diseases, such as Alzheimer's and Parkinson's^35^. Othman et al.^36^ evidenced that the benefits of the honey, due to its antioxidant activity, and to its stimulating effects in increasing the levels of the brain-derived neurotrophic factors and the acetylcholine concentrations, and concurrently the decrease of the AChE activity. Such positive effects were attributed by the authors to the presence of polyphenols. More recently, Szwajgier et al.^37^ evidenced the potential benefits of nineteen types of honey, which can represent an important font of cholinesterase inhibitors, playing a role in the Alzheimer disease. Honey also inhibits the growth of pathogens, which presence and diffusion can damage the Central Nervous System. In this study, the contents of total polyphenols (TP), total flavonoids, and vitamin C, present in six *Citrus* honeys (bergamot, clementine, lemon, lime, orange, and tangerine) were determined. The anti-inflammatory and antioxidant activities, as well as their inhibitory effects on three enzymes involved in NDDs (acetylcholinesterase, butyrylcholinesterase, and tyrosinase), were also evaluated. Finally, the biofilm inhibitory activity was assessed against some bacteria involved in NDDs. Results are shown in Tables 1, 2, 3, 4.

**Table 1:** Total polyphenol content (TPC), total flavonoids content, vitamin C content, in *vitro* anti-inflammatory activity, antioxidant activity, cholinesterases and tyrosinase inhibitory activity of the six honeys.

	Bergamot	Clementine	Lemon	Lime	Orange	Tangerine
TPC (*µ*g GAE/g sample)	119.9 ± 8.70^b^	143.47 ± 5.90^a^	161.75 ± 9.40^a^	164.32 ± 3.80^a^	108.6 ± 7.60^b^	126.37 ± 2.30 ^a^
Flavonoids (*µ*g RE/g sample)	76.40 ± 7.81^a^	81.09 ± 8.58^a^	165.83 ± 3.46^c^	53.20 ± 4.45^b^	29.86 ± 7.64^b^	124.88 ± 8.68^c^
Vitamin C (mg/100gr)	8.40 ± 0.30^b^	25.18 ± 0.50^c^	6.30 ± 0.30^b^	37.30 ± 1.50^d^	8.70 ± 0.30^b^	7.40 ± 0.20^b^
ABTS• (*µ*molTE/g sample)	1.79 ± 0.04^a^	2.38 ± 0.96^a^	4.57 ± 0.22^a^	7.39 ± 2.33^a^	1.84 ± 0.03^a^	1.49 ± 0.06^a^
Inhibitory effect against the bovine serum albumin degradation (EC_50_, mg) *	73.87 ± 23.04^b^	n.d	48.47 ± 8.42^b^	63.18 ± 15.22^b^	71.64 ± 19.71^b^	105.25 ± 22.11^b^
AChE* inhibition (%)	8.49 ± 0.20	5.03 ± 1.30	7.70 ± 0.70^c^	3.50 ± 0.40^c^	5.06 ± 1.10^c^	12.04 ± 0.80^c^
AChE* inhibition (IC_50_,mg)	137.40^a^	212.20^a^	101.7^a^	245.30^b^	227.80^b^	116.90^a^
BChE* inhibition (%	19.11 ± 3.50^c^	10.65 ± 2.10^c^	n.d	n.d	7.11 ± 1.30^c^	18.03 ± 0.30^c^
BChE* inhibition (IC_50_,mg)	57.81^b^	100.29^a^	n.d	n.d	161.47^b^	79.89^a^
Tyrosinase activity inhibition (L-tirosine %) *	82.30 ± 1.90^b^	72.60 ± 2.80^b^	94.10 ± 5.10^b^	85.60 ± 1.70^b^	82.60 ± 4.40^b^	73.8 ± 2.30^b^
Tyrosinase activity inhibition DOPA (IC_50_, mg)*	19.02 ± 0.90^c^ ±	16.19 ± 1.50^c^	11.85 ± 1.2^c^	8.18 ± 0.80^c^	20.96 ± 2.10^b^	24.44 ± 1.10^b^

Results are expressed as the average of three independent experiments. RE: Rutin equivalent GAE: Gallic acid equivalent. * a: *p* <  0.5; b: *p* <  0.01; c: *p* <  0.001; d: *p* <  0.0001 compared to the positive control; a: *p* <  0.5; b: *p* <  0.01; c: *p* <  0.001; d: *p* <  0.0001 compared to the average data (ANOVA followed by Dunnett’s multiple comparison test), n.d. = not detected.

### Polyphenols, flavonoids, and vitamin C content

As indicated in Table 1, the TPC was always higher than 100 μg/g of the fresh product, ranging between 108.91 (orange honey) and 164.32 μg/g^-^(lime honey). The average amount of TPC fits with the data reported by other authors, such as Perna et al.^38^, who, analyzing eighteen samples of *Citrus* honey, observed that the TPC was about 12.15 mg/100 g. The amounts we found was overall lower than that of Homrani et al.^39^, whose analysis, carried out on five samples of *Citrus* honey (the species was not indicated), revealed a TP content of 50.5 mg/100 g of the product. Di Petrillo et al.^40^ found, in a *Citrus* honey, a TPC of 41.64 mg /100 g of the product. Imtara et al.^41^ found a similar amount in a lemon honey. However, unlike the latter samples, the honeys we analyzed contained mainly flavonoids, the quantity of which, reached no less than 29 μg/g (orange honey). Considering the recommended amount of honey consumption, the intake of flavonoids can be even over 1.658 mg, present in 10 g of lemon honey, corresponding to the consumption of half dose of honey in a week^42^. The amount of Vitamin C emerged as highly variable in the six honeys, ranging between 6.3 (lemon honey) up to 37.30 mg /100 of the fresh product (lime honey). These values were higher than those found by Perna et al.^43^, and Ciulu et al.^44^, although both researchers indicated the generic term of *Citrus* without indicating the species.

### Antioxidant activity of the honey and its inhibitory effect against the bovine serum albumin degradation

Several studies evidenced that dietary plant polyphenols show powerful neuro-protective effects in different NDDs^45^ and their antioxidant activity^46^. The onset of neurodegenerative diseases is also due to an exacerbation of inflammatory processes, giving rise also to an increase of protein denaturation, and membrane alteration. This leads to a release of cytokines, prostaglandins, and histamine, chemical messengers that determines the occurrence of the chemotaxis, a body's natural defense mechanism^47^. Except for clementine honey, the honeys tested in our experiments exhibited a capacity to protect the BSA against denaturation. Inhibition at 50% (IC_50_) ranged between 48.47 mg (lemon honey) to 105.25 mg (tangerine honey). Diclofenac, the standard anti-inflammatory drug used as control, showed an IC_50_ value of 5.475 mg. Other scientists reported a higher anti-inflammatory activity, but calculated respect to acetylsalicylic acid^47^. IC_50_ values indicated that the *Citrus* honeys studied might provide good anti-inflammatory protection, considering that a teaspoon of honey contains about 2 g of the product. By the analysis of correlation, the protective effect of *Citrus* honey against the inflammatory activity seemed to be due to the TP content (Corr-Coeff = − 0.566), and less to Vitamin C (Corr-Coeff =  −0.277). Results were in agreement with most of the monofloral honeys analyzed by Ruiz et al., where the content of Vitamin C was also correlated to the honey antioxidant effect^48^. Besides its antioxidant properties, studies have shown that Vitamin C possess neuroprotective properties and can neutralize superoxide radical^49^. The *Citrus* honeys studied in this research exhibited a variable antioxidant trend. TEAC values varied between 1.485 µmol TEAC / g (tangerine honey, with the weakest antioxidant activity) and 7.385 µmol TEAC / g of the product (lime honey, the strongest among those analyzed). These data were, in some cases, very similar to those observed by Al-Qahtani et al. in some Saudi Arabia honeys^50^.

### Cholinesterase inhibitory action

In the developmental analysis of such neurodegenerative diseases, such as Alzheimer disease, the concentrations of acetylcholine and choline are rapidly declining, while the AChE enzyme is excessively increased, leading to cellular nerve dysfunction^18^. Thus, a concurrent decrease of the cholinergic or neurotransmitter activity in the central nervous system is activated, with a subsequent loss of brain memory and cognitive function^51^. The possible inhibition of cholinesterase with possible obstacles to the potential breakdown of cholinergic synapses can be considered likely prevention and treatment of AD. Many studies on brain function have already been done, but the majority of these works had focused on γ-aminobutyric acid (GABA), the primary inhibitory neurotransmitter of the Central Nervous System, including also the effect of some natural compounds^52^. In our study, the in vitro prospective inhibitory effect of the six *Citrus* types of honey on the ACHe and BCHe was investigated. Results are shown in Table 1. All samples inhibited the AChE activity. The best inhibitory performances were exhibited by the tangerine, bergamot, and lemon honeys, with IC_50_ values ranging between 101.7 mg (lemon honey) and 137.4 mg (bergamot honey). The other three types of *Citrus* honey, although active, exerted an inhibition of about a half (ranging between IC_50_ = 212.2 mg and IC_50_ = 245.3 mg, for clementine and lime honey, respectively). The inhibitory activity of the honeys on the BChE resulted more incisive, although not given by all the studied honeys. Once again, tangerine honey (IC_50_ = 79.89 mg) and mainly bergamot honey (IC_50_ = 57.81 mg) were the most active BChE inhibitors. Lemon honey, which even resulted active against the AChE, in this case, resulted completely ineffective, as lime honey. Different papers reported the cholinesterase inhibitory activity exhibited by the honey through in vitro studies. Baranowska-Wójcik et al.^35^ analyzed 47 kinds of Polish honey, finding an AChE inhibitory activity reaching even 39.06%. The results also confirmed that the behavior discrepancy found among the types of honey could be due to the different botanical origins of the products. The antioxidant and inhibitory AChE activity of Malaysian Borneo^53^ and Algerian honeys^54^ demonstrated in most cases a correlation between the capacity of these honeys to inhibit the AChE and their antioxidant activity, as well as the content of polyphenols. Loizzo et al.^55^ studied the cholinesterase inhibitory activity of fig honey and found AChE and BChE IC_50_ values of 46.0 and 46.8 µg ml^−1^, respectively. The investigation of the activity of BChE is of clear relevance since in the late stages of the Alzheimer’s disease, levels of AChE decline by up to 85% and BChE becomes the predominant cholinesterase in the brain^56^.

### Tyrosinase inhibitory activity

The neurodegenerative process occurs with an excessive presence of tyrosinase, which overexposure presumably determines the onset of PD^19^ when a threshold value of neuromelanin is exceeded. Thus, the reduction of intracellular neuro-melanin, also through the inhibition of tyrosinase, could prevent the neurodegeneration, concurring to avoid or at least to retard or limit the occurrence and gravity of PD. We assessed the tyrosinase inhibitory activity first using tyrosine (expressed in percentage), and then through the l-DOPA as substrate and expressing the results in terms of IC_50_. As a positive control, we used kojic acid, one of the most known tyrosinase inhibitors, used to treat some skin disorders^57^. In the test carried out with tyrosine, all types of honey evidenced an excellent capacity to act as tyrosinase inhibitors, with percentages of inhibition ranging between 72% (clementine honey) and 94.1% (lemon honey). Interestingly, in the DOPA test, the IC_50_ values did not exceed 24.44 mg, and lime honey exhibited the best anti-tyrosinase activity, with an IC_50_ value of 8.18 mg. Jantakee et al.^58^ studied the activities of different types of Thay honey, such as coffee flower honey. They observed that, in some cases, the tyrosinase enzyme's inhibitory activity was 63.46%. More recently, Habib et al.^59^ demonstrated that at least a type of honey from arid land could act against tyrosinase activity and concurrently prevent protein damage. The reaction of inhibition exhibited by the honey could be dependent also on the hydroxyl groups of the phenolic compounds^60^. The mechanism of antioxidant activity may also be an essential aspect of the inhibition too^61^. The literature about the ACHe, BCHe, and tyrosinase inhibitory activity of honey are few. Moreover, it seems that there are no studies that have evaluated concurrently the inhibitory properties exhibited by the honey of these six *Citrus* species, on all three enzymes involved in neurodegenerative processes, nor the inhibitory activity of *Citrus* honey against tyrosinase was assessed, using both tyrosine and l-DOPA.

The correlation analysis indicated some interesting aspects: the inhibitory activity exhibited by the honey against the action of cholinesterases seemed linked to each other, as it is possible see from the correlation results. Considering the percentages of inhibitory activity, the Corr-Coeff value was = 0.836; on the other hand, considering the inhibitory activity expressed as IC_50_, we found a strong correlation (Corr-Coeff =  −0.823) too. Both the inhibitory cholinesterases activity (expressed as IC_50_) exhibited by the six *Citrus* honeys seemed, in turn, to be directly related to the amount of total flavonoids (Corr-Coeff =  −0.860 for AChE; Corr-Coeff =  −0.738 for BChE inhibitory activity). Similarly, when we the inhibitory activity was expressed as a percentage, the Corr-Coeffs were 0.616 and 0.744 for AChE and BChE, respectively. The TEAC antioxidant activity, in turn, was due to the total polyphenols (Corr-Coeff = 0,846) and the vitamin C (Corr-Coeff = 0.702) content. Furthermore, intriguingly, TEAC activity could be related to the anti-inflammatory activity (Corr-Coeff =  −0.607). In addition, a strong correlation between TEAC and tyrosinase inhibitory activity on DOPA (Corr-Coeff =  −0.925) was registered. From our data, the anti-inflammatory activity correlated with the inhibition of tyrosinase on DOPA (Corr-Coeff = 0.804) and on L-tyrosine (Corr-Coeff =  −0,974) indeed. Finally, tyrosinase inhibition performed on DOPA and expressed as IC_50_ showed a direct correlation with vitamin C but, above all, with TP (Corr-Coeff =  −0.874). These findings suggest that total polyphenols and vitamin C contents influence, although not directly, also the anti-inflammatory action, related to the antioxidant activity and to the inhibition of tyrosinase (on the DOPA substrate).

### Anti-biofilm activity

Table 2 shows the MIC of the *Citrus* honeys, determined to evaluate then the inhibitory action on the bacterial ability to form a biofilm (Table 3) and on the metabolism of sessile cells (Table 4) within the biofilm. The results showed that, with the sole exception of lime honey (ineffective at 10 µg/ml *vs. A. baumannii* and *L. monocytogenes*), the *Citrus* honeys managed to inhibit all the tested bacteria used as models of pathogens involved in neurodegenerative diseases (Table 3). The inhibition of biofilm formation was between 4.30% (10 µg/ml of lime honey *vs. S. aureus*) and 89.41% (20 µg/ml of lime honey *vs. L. monocytogenes*). All the bacteria were sensitive to the presence of *Citrus* honeys. *A. baumannii* resulted susceptible to all types of *Citrus* honey (except lime honey), with an inhibitory activity on biofilm formation not less than 56.69% (10 µg/ml of clementine honey). This bacterium was susceptible, at 20 µg/ml, to the honeys of orange (82.99% inhibition), bergamot (87.70%), clementine (79.92%), lemon (79.23%). In the presence of the tangerine honey, the inhibition reached 72.29%. Therefore, the lower honey concentration caused inhibition percentages of *A. baumannii* up to 76.44% (lemon honey) and even 81.06% (bergamot honey). The biofilm inhibitory action of all six types of *Citrus* honey was effective against *E. coli*, with inhibition rates ranging between 33.85% (10 µg/ml of clementine honey) and 87.44% (20 µg/ml of lime honey). Furthermore, an inhibitory action against *L. monocytogenes* was observed, with percentages of 89.45%- 89.47% (lime and clementine honey, respectively). The inhibitory biofilm efficacy *vs.* such bacteria was higher than *vs A. baumannii*, as the percentage of inhibition never dropped below 60% (with 10 µg/ml of orange honey). The inhibition against *E. coli* and *L. monocytogenes* seemed, from the MTT test, to be essentially due to an activity of honeys on the metabolism of bacterial cells (Table 4). In fact, for example, a biofilm-inhibitory action exerted by the clementine honey *vs. L. monocytogenes* (89.47%) was echoed by a similar inhibitory action (90.27%) on the metabolism of the sessile cells of this microorganism. A similar effect was observed when the tangerine honey acted against *E. coli* (91.12%). The inhibitory biofilm action exerted by the *Citrus* honeys against *A. baumannii, P. aeruginosa*, and *S. aureus* did not seem to correspond to an activity on bacterial metabolism. *P. aeruginosa*, which also underwent an inhibitory biofilm action in the presence of all honeys, was almost insensitive to the honey action on metabolism of its sessile cells, so that we observed a maximum of 19.19% inhibition. *A. baumannii* exhibited a similar trend: the metabolic-inhibitory percentage reached just 13.24%. *S. aureus*, which also showed a remarkable sensitivity to the presence of *Citrus* honeys (with inhibition percentages that reached 80.10%), was utterly insensitive in the MTT test. This could support the hypothesis that the mechanisms leading to the inhibition of the biofilm do not necessarily pass through an inhibitory metabolic action fighting the sessile cells, but that could be probably due to other mechanisms of a non-metabolic nature, such as the capacity of the honey to damage the bacterial membrane, the action on bacterial nucleic acid, as well as to the presence in the honey of some enzymes, such as catalase, or to the content and type of polyphenols, which activity on biofilm and metabolism of biofilm has been demonstrated^27^. Therefore, the results show that these types of *Citrus* monofloral honey could exert a versatile biological activity still poorly studied. Their action against pathogens might be considered interesting also from the point of view of neurodegenerative pathologies. Honey affected the virulence of pathogens, and the degradation of BSA, an useful in vitro, and preliminary test that could give useul first information on the inflammatory pathway caused also by bacterial infections or by a disbalance of the gut microbiota^62,63^. However, this hypothesis needs to be demonstrated with studies on humans, using the suggested doses (20 g/week) that does not give a negative effect on the health (for example, an increase of the glycemic level). The activity exhibited by honeys *vs. E. coli*, *P. aeruginosa* (and, in the case of bergamot honey, *vs S. aureus*) indicates that these honeys can limit onset, proliferation, and virulence of microorganisms whose role in neurodegenerative diseases is becoming increasingly evident^64^.

**Table 2:** Minimal inhibitory concentration (MIC, *µ*g/mL) of the monofloral *Citrus* honeys, needed to block the metabolic activity of the five bacterial strains, evaluated through the resazurin test.

	*A. baumannii*	*E. coli*	*L. monocytogenes*	*P. aeruginosa*	*S. aureus*
Bergamot	30 (± 2)	35 (± 3)^c^	30 (± 2)	30 (± 2)	35 (± 4)
Clementine	35 (± 3)	40 (± 5)^d^	30 (± 5)	35 (± 5)	45 (± 5)^b^
Lemon	30 (± 3)	30 (± 2)	35 (± 2)	35 (± 4)	35 (± 2)
Lime	50 (± 2)^d^	35 (± 4)^c^	50 (± 2)^d^	40 (± 3)	45 (± 3)^b^
Orange	35 (± 3)	40 (± 4)^d^	35 (± 2)	35 (± 3)	40 (± 4)
Tangerine	35 (± 2)	40 (± 2)^d^	50 (± 2)^d^	30 (± 2)	45 (± 3)^b^
Tetracycline	30 (± 2)	25 (± 3)	33 (± 2)	34 (± 2)	36 (± 2)

Results are expressed as the mean of three experiments ± SD. a: *p* <  0.5; b: *p* <  0.01; c: *p* <  0.001; d: *p* <  0.0001 compared with the tetracycline used as control (ANOVA followed by Dunnett’s multiple comparison test).

**Table 3:** Inhibitory activity of *Citrus* monofloral honey on the bacterial biofilm formation. Samples were tested at two concentrations, 10 µg/ml and 20 µg/ml against pathogens *A. baumannii*, *E. coli*, *L. monocytogenes*, *P. aeruginosa*, and *S. aureus* following the method described in the section of material and methods.

	*A. baumannii*	*E. coli*	*L. monocytogenes*	*P. aeruginosa*	*S. aureus*
Bergamot 10 µg/ml	58.59^d^ (± 0.29)	38.03^d^ (± 4.04)	60.09^d^ (± 1.46)	52.49^d^ (± 1.11)	25.91^d^ (± 0.86)
Bergamot20 µg/ml	82.99^d^ (± 0.72)	56.10^d^ (± 1.19)	78.96^d^ (± 0.72)	69.61^d^ (± 1.53)	40.32^d^ (± 1.11)
Clementine 10 µg/ml	81.06^d^ (± 0.90)	50.31^d^ (± 0.95)	78.53^d^ (± 1.25)	68.08^d^ (± 1.53)	49.15^d^ (± 0.61)
Clementine 20 µg/ml	87.70^d^ (± 0.83)	77.31^d^ (± 0.81)	79.55^d^ (± 0.80)	72.33^d^ (± 1.10)	80.10^d^ (± 1.09)
Lemon 10 µg/ml	56.69^d^ (± 0.75)	33.85^d^ (± 5.94)	74.33^d^ (± 0.58)	52.94^d^ (± 0.55)	16.48^d^ (± 0.82)
Lemon 20 µg/ml	79.92^d^ (± 1.97)	43.01^d^ (± 0.56)	89.47^d^ (± 0.51)	60.66^d^ (± 0.71)	20.74^d^ (± 1.46)
Lime 10 µg/ml	0.00 ± 0.00	52.78^d^ (± 2.94)	0.00 ± 0.00	12.49^d^ (± 0.59)	4.30^b^ (± 0.84)
Lime 20 µg/ml	68.97^d^ (± 1.56)	87.44^d^ (± 0.66)	89.45^d^ (± 0.77)	69.17^d^ (± 0.84)	36.04^d^ (± 0.56)
Orange 10 µg/ml	76.44^d^ (± 0.34)	70.00^d^ (± 1.51)	79.80^d^ (± 3.69)	52.80^d^ (± 0.34)	17.39^d^ (± 1.19)
Orange 20 µg/ml	79.23^d^ (± 1.12)	72.01^d^ (± 0.97)	86.09^d^ (± 0.64)	60.11^d^ (± 0.87)	44.55^d^ (± 0.84)
Tangerine 10 µg/ml	59.63^d^ (± 0.93)	38.32^d^ (± 0.97)	0.00 ± 0.00	65.71^d^ (± 0.86)	5.58^d^ (± 0.71)
Tangerine 20 µg/ml	72.29^d^ (± 0.82)	52.68^d^ (± 0.90)	84.48^d^ (± 0.90)	73.97^d^ (± 0.24)	34.84^d^ (± 1.93)

The percentages of inhibition were calculated assuming for the control (untreated bacteria) an inhibition = 0. Results are expressed as the mean of three experiments (± SD). a: *p* <  0.5; b: *p* <  0.01; c: *p* <  0.001; d: *p* <  0.0001 (ANOVA followed by Dunnett’s multiple comparison test).

**Table 4:** Inhibitory activity of *Citrus* honey on the bacterial cell metabolism within biofilm, tested at concentrations of 10 µg /ml and 20 µg /ml.

	*A. baumannii*	*E. coli*	*L. monocytogenes*	*P. aeruginosa*	*S .aureus*
Bergamot 10 µg/ml	0.00 ± 0.00	89.77^a^ ± 0.01	73.87^a^ ± 0.51	0.00 ± 0.00	0.00 ± 0.00
Bergamot 20 µg/ml	13.24^a^ ± 0.10	90.16 ± 0.05^a^	87.30^a^ ± 0.21	1.32 ± 0.04	0.00 ± 0.00
Clementine 10 µg/ml	0.00 ± 0.00	86.31^a^ ± 0.11	89.46^a^ ± 0.02	0.00 ± 0.00	0.00 ± 0.00
Clementine 20 µg/ml	0.00 ± 0.00	89.57^a^ ± 0.07	90.27^a^ ± 0.03	8.55^a^ ± 0.04	0.00 ± 0.00
Lemon 10 µg/ml	0.00 ± 0.00	82.37^a^ ± 0.11	78.85^a^ ± 0.29	0.00 ± 0.00	0.00 ± 0.00
Lemon 20 µg/ml	0.00 ± 0.00	86.25^a^ ± 0.67	82.21^a^ ± 0.65	0.00 ± 0.00	0.00 ± 0.00
Lime 10 µg/ml	0.00 ± 0.00	58.82^a^ ± 0.23	0.00 ± 0.00	0.00 ± 0.00	0.00 ± 0.00
Lime 20 µg/ml	0.00 ± 0.00	83.88^a^ ± 0.11	74.63^a^ ± 0.23	19.19^a^ ± 1.67	0.00 ± 0.00
Orange 10 µg/ml	0.00 ± 0.00	62.86^a^ ± 0.81	69.26 ± 0.80 ^a^	0.00 ± 0.00	0.00 ± 0.00
Orange 20 µg/ml	0.00 ± 0.00	85.89^a^ ± 0.93	79.18 ± 0.40^a^	0.00 ± 0.00	0.00 ± 0.00
Tangerine 10 µg/ml	0.00 ± 0.00	82.97^a^ ± 0.37	0.00 ± 0.00	0.00 ± 0.00	0.00 ± 0.00
Tangerine 20 µg/ml	11.68^a^ ± 0.41	91.12^a^ ± 0.11	79.62^a^ ± 0.89	2.78 ± 0.11	0.00 ± 0.00

The percentage of inhibition was calculated assuming for the control (untreated bacteria) an inhibition = 0. Results are expressed as the mean of three experiments (± SD). a: *p* <  0.0001 (ANOVA followed by Dunnett’s multiple comparison test).

The *Citrus* honey studied have shown interesting potential biological properties. Their inhibiting effect against some enzymatic activities involved in NDDs makes them attractive potential candidates to support, in the presence of an appropriate diet or in a pharmacological treatment, some NDDs fighting especially elderly people, so that honey, already considered as healthy food, could represent a further added value for the diet. The results from the microbial tests indicated the extensive action of these honeys against different pathogens. Remarkable is the action of the honey on the biofilm and on the metabolism of biofilm, a complex situation to contrast, which not always is shown by natural compounds with antimicrobial activity, especially in fragile people, which immune defenses are notoriously weaker and more susceptible to the microbial attack. Future studies will try to better define the role of the honey also with test on cells.

## Conclusions

Future steps will be to determine the phenolic composition and the potential correlation with the functional activity of the honeys, not only taking into consideration NDDs but also other pillar enzymes involved in dismetabolic pathologies, such as the α-glucosidase and the α-amylase activity. The results will be useful in the definition of the in vivo activity of these types of honey on human health.

## Data Availability

The datasets used and/or analyzed during the current study are available from the corresponding author on reasonable request.
